# Conversion and validation of rectal constraints for prostate carcinoma receiving hypofractionated carbon-ion radiotherapy with a local effect model

**DOI:** 10.1186/s13014-021-01801-w

**Published:** 2021-04-13

**Authors:** Weiwei Wang, Ping Li, Yinxiangzi Sheng, Zhijie Huang, Jingfang Zhao, Zhengshan Hong, Kambiz Shahnazi, Guo-Liang Jiang, Qing Zhang

**Affiliations:** 1 Department of Medical Physics, Shanghai Proton and Heavy Ion Center, Shanghai Key Laboratory of Radiation Oncology (20dz226100), Shanghai Engineering Research Center of Proton and Heavy Ion Radiation Therapy, 4365 Kangxin Road, Pudong District, Shanghai, 201315 China; 2 Department of Radiation Oncology, Shanghai Proton and Heavy Ion Center; Shanghai Key Laboratory of Radiation Oncology (20dz226100), Shanghai Engineering Research Center of Proton and Heavy Ion Radiation Therapy, 4365 Kangxin Road, Pudong District, Shanghai, 201315 China; 3 Department of Radiation Oncology, Fudan University Shanghai Cancer Center, Xuhui District, 270 Dongan Road, Shanghai, 200032 China

**Keywords:** Carbon ion radiotherapy, MKM, LEM, Hypofractionated CIRT, Prostate carcinoma

## Abstract

**Background:**

The study objective was to establish the local effect model (LEM) rectum constraints for 12-, 8-, and 4-fraction carbon-ion radiotherapy (CIRT) in patients with localized prostate carcinoma (PCA) using microdosimetric kinetic model (MKM)-defined and LEM-defined constraints for 16-fraction CIRT.

**Methods:**

We analyzed 40 patients with PCA who received 16- or 12-fraction CIRT at our center. Linear-quadratic (LQ) and RBE-conversion models were employed to convert the constraints into various fractionations and biophysical models. Based on them, the MKM LQ strategy converted MKM rectum constraints for 16-fraction CIRT to 12-, 8-, and 4-fraction CIRT using the LQ model. Then, MKM constraints were converted to LEM using the RBE-conversion model. Meanwhile the LEM LQ strategy converted MKM rectum constraints for 16-fraction CIRT to LEM using the RBE-conversion model. Then, LEM constraints were converted from 16-fraction constraints to the rectum constraints for 12-, 8-, and 4-fraction CIRT using the LQ model. The LEM constraints for 16- and 12-fraction CIRT were evaluated using rectum doses and clinical follow-up. To adapt them for the MKM LQ strategy, CNAO LEM constraints were first converted to MKM constraints using the RBE-conversion model.

**Results:**

The NIRS (i.e. D_MKM_|v, V-20%, 10%, 5%, and 0%) and CNAO rectum constraints (i.e. D_LEM_|v, V-10 cc, 5 cc, and 1 cc) were converted for 12-fraction CIRT using the MKM LQ strategy to LEM 37.60, 49.74, 55.27, and 58.01 Gy (RBE), and 45.97, 51.70, and 55.97 Gy (RBE), and using the LEM LQ strategy to 39.55, 53.08, 58.91, and 61.73 Gy (RBE), and 49.14, 55.30, and 59.69 Gy (RBE). We also established LEM constraints for 8- and 4-fraction CIRT. The 10-patient RBE-conversion model was comparable to 30-patient model. Eight patients who received 16-fraction CIRT exceeded the corresponding rectum constraints; the others were within the constraints. After a median follow-up of 10.8 months (7.1–20.8), No ≥ G1 late rectum toxicities were observed.

**Conclusions:**

The LEM rectum constraints from the MKM LQ strategy were more conservative and might serve as the reference for hypofractionated CIRT. However, Long-term follow-up plus additional patients is necessary.

## Background

Radiotherapy is a curative treatment for localized prostate carcinoma (PCA). Advanced techniques like intensity-modulated radiotherapy or volume-modulated arc therapy have increased treatment safety of photon radiotherapy [1]; however, irradiation doses remain limited by the tolerance of surrounding organs at risk (OARs). Proton or carbon-ion radiotherapy (CIRT)—which feature reduced radiation of the rectum and bladder—might be more effective and safe, especially for hypofractionated CIRT [2].

In 1995, the National Institute for Quantum and Radiological Science and Technology—the former National Institute of Radiobiological Science (NIRS) in Japan—implemented the first CIRT protocol using 66–60 Gy (RBE) in 20-fractions for PCA [3]. To further improve the efficacy and safety, NIRS administered 57.6 Gy (RBE) in 16-fractions, equivalent to 63 Gy (RBE) in 20-fractions, based on a linear-quadratic (LQ) model [4]. Using a similar approach, the rectal constraints were also converted to 16-fractions. They found that the local control was the same as before, while late toxicity (≥ G1) was further reduced to be < 10%. Encouraged by the aforementioned benefits, NIRS developed a 12-fraction protocol [5]. Currently, a 4-fraction clinical trial is ongoing.

NIRS published dose constraints for the rectum using the microdosimetric kinetic model (MKM) [6]. However, the MKM [7] and local effect model (LEM) [8] hold different model assumptions, refer to different endpoints and cells, et cetera. [9]. Thus, LEM centers had to convert NIRS doses when referring to their experience [10, 11]. Our previous study converted the MKM experience to LEM [12] and successfully applied the converted constraints to our dose-escalation clinical trial, which examined dose escalations to 4.10 Gy (RBE) per fraction by 16 fractions. The Centro Nazionale di Adroterapia Oncologica (CNAO) converted the NIRS experience to LEM and summarized their own experiences, eventually finalizing their own rectum constraints [13]. All the aforementioned constraints were for 16-fraction protocol. Starting in September, 2019, our center has implemented a new 12-fraction protocol. The major issue for performing shorter course protocols—even the 4-fraction protocol—is how to impose feasible rectum dose constraints.

The LQ model [14] has been widely used in photon radiotherapy to convert doses between different fractionations, based on biological equivalent doses. Our previous study [12] established an RBE-conversion model to convert RBE-weighted doses with MKM (MKM doses) to the RBE-weighted dose with LEM (LEM doses). In this study, to use the CNAO experience for our shorter course CIRT, we first examined the agreement between CNAO constraints and our constraints for 16-fraction therapy. Then, based on the LQ model and the RBE-conversion model, two strategies were used to convert both the NIRS and CNAO rectum constraints for 16-fraction CIRT to LEM constraints for 12-, 8-, and 4-fraction CIRT. We additionally followed 40 previously treated patients to validate the established constraints.

## Methods

### Patients’ information

Forty patients with PCA, who received 16- or 12-fraction CIRT at our center from October 2018 to January 2020, were selected from our clinical database. Patients’ characteristics are shown in Table 1. The risk profiles were grouped based on the initial PSA, imaging, and pathology information. The clinical target volume (CTV) included the whole prostate and seminal vesicles and was based on the different risk groups. The planning target volume was expended based on the CTV by adding 10.0-mm margins laterally and 5.0-mm margins in other directions. The OARs (e.g., bladder and rectum) were contoured according to the RTOG normal tissue guidelines [15]. Syngo (V13B, Siemens, Germany) with a LEM was used for all clinical treatment planning. Thirty-eight patients received 16-fraction CIRT with a 4.00 Gy (RBE) to 4.10 Gy (RBE) dose-per-fraction and two patients received 12-fraction CIRT with a dose-per-fraction of 4.50 Gy (RBE). Rectum dose-volume histograms (DVH) from Syngo were collected for validation.

**Table 1:** Patients’ characteristics

Parameter	Total N = 40
Gleason score	
6	8 (20.0%)
7	12 (30.0%)
8–10	20 (50.0%)
T stage (AJCC 8^th^)	
T2	33 (82.5%)
T3	5 (12.5%)
T4	1 (2.5%)
^a^Tx	1 (2.5%)
Risk group	
Low	2 (5.0%)
Intermediate	14 (35.0%)
High	23 (57.5%)
^a^Unknown	1 (2.5%)
Initial PSA	
< 10 ng/mL	16 (40.0%)
10–19.9 ng/mL	13 (32.5%)
≥ 20 ng/mL	11 (27.5%)
Parameter	Volume (cc)
CTV/median (range, cc)	92.85 (48.08–164.94)
≤ 100.00	68.44 (48.08–94.5) (52.5%)
> 100.00	121.37 (107.42–164.94) (47.5%)
Rectum/median (range, cc)	50.66 (34.59–75.29)
≤ 50.00	44.04 (34.59–49.89) (47.5%)
> 50.00	56.19 (50.41–75.29) (52.5%)

^a^One patient received transurethral prostatic resection (TURP) before CIRT, therefore, his T stage and risk profile are unknown

### Study framework

**Fig. 1:**
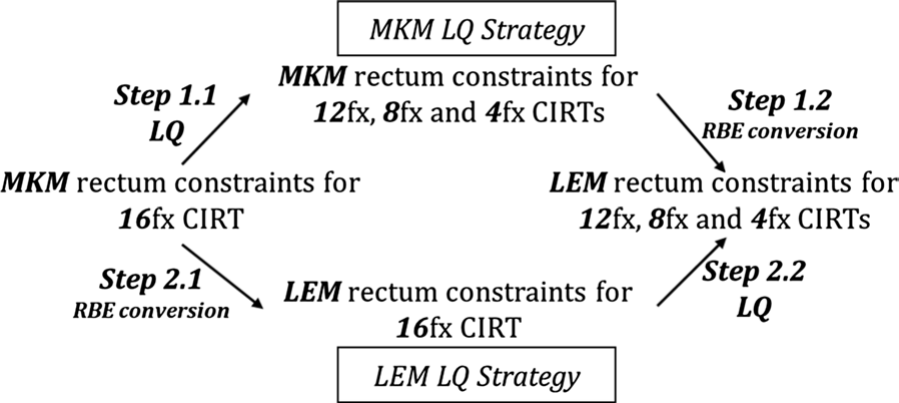
Study framework

Figure 1 describes the study framework. Two strategies were employed: MKM LQ and LEM LQ.

For the MKM LQ strategy, in Step 1.1 (LQ), the MKM rectum constraints for 16-fraction CIRT were converted to the MKM rectum constraints for 12-, 8-, and 4-fraction CIRT based on the LQ model. The α/β ratio of 3.9 Gy to the rectum was the same as the one used by Uhl et al. [16]. In Step 1.2 (RBE conversion), three MKM rectum constraints were converted to the LEM rectum constraints for 12-, 8-, and 4-fraction CIRT using the RBE-conversion model.

For the LEM LQ strategy, in Step 2.1 (RBE conversion), the MKM rectum constraints for 16-fraction CIRT were converted to the LEM rectum constraints for 16-fraction CIRT using the RBE-conversion model. In Step 2.2 (LQ), the LEM rectum constraints for 16-fraction CIRT were converted to the LEM rectum constraints for 12-, 8-, and 4-fraction CIRT using the LQ model.

### RBE-conversion model and LQ model

Our previous study [12] established an RBE-conversion model for 16-fraction CIRT. We additionally developed three RBE-conversion models respectively for 12-fraction CIRT with MKM prescriptions 5.3 Gy (RBE)/fx, for 8-fraction CIRT with MKM prescription 7.0 Gy (RBE)/fx, and for 4-fraction CIRT with MKM prescription 10.0 Gy (RBE)/fx. These were done with our research treatment planning system, Raystation (8A, Raysearch, Sweden). We randomly selected 10 of the 40 enrolled patients. Their planning CT and contouring from Syngo were exported to the Raystation. The process of generating a RBE-conversion model was described briefly in the following. The MKM plans were generated and optimized to fulfill the MKM prescriptions. Then, the LEM plans were generated by recalculating the physical doses from MKM plans. Based on the isodose volumes from the MKM and LEM plans, a conversion model for converting the MKM doses to LEM doses was established.

For the LQ model, the following equation was used:

$$N_{2} {*}d_{2} {*}\left( {1 + d_{2} /\left( {\alpha /\beta } \right)} \right) = N_{1} {*}d_{1} {*}\left( {1 + d_{1} /\left( {\alpha /\beta } \right)} \right)$$

where α/β is 3.9 Gy, *d* is the dose-per-fraction, and *N* is the total fractionation.

### Rectum constraints for 16-fraction CIRT

NIRS published their rectum dose constraints for 16-fraction CIRT [17, 18] with MKM, which were percentage volume constraints (i.e., D_MKM|V,_ V-20%, 10%, 5%, and 0% were ≤ 28.80, 46.40, 56.00, and 60.80 Gy (RBE)). CNAO [13] first reviewed NIRS constraints (i.e., D_MKM|V,_ V-20%, 10%, 5%, and 1% were ≤ 28.80, 46.40, 60.00, and 66.00 Gy (RBE)), then converted them to LEM. Based on that, CNAO summarized their LEM experience and finalized their own absolute volume constraints (i.e., D_LEM|v_, V-10 cc, 5 cc, and 1 cc were ≤ 54.00, 61.00, and 66.00 Gy (RBE)). We first examined the agreement between CNAO’s study and our previous studies by comparing the respectively converted LEM doses from the same NIRS constraints. Then, based on the agreement, CNAO absolute volume constraints were converted to the LEM constraints for 12-, 8-, and 4-fraction CIRT based on our two strategies. To perform MKM LQ strategy on the CNAO constraints, their LEM constraints were first converted backward to the MKM constraints, then re-converted to the final LEM constraints based on our RBE-conversion model.

### Conversion uncertainty

The LQ model is an analytical approach. However, the RBE-conversion model was based on only 10 patients. To evaluate its conversion uncertainty, the other 30 patients were used to generate a new RBE-conversion model for 16-fraction CIRT and compared to the counterpart, which based on 10 patients.

### Clinical evaluation

Clinical follow-up data were collected and compared to the LEM rectum constraints for 16- and 12-fraction CIRT. Patients were followed-up by a radiation oncologist at one month, then every three months after CIRT completion for the first two years, every six months over the next three years, and yearly afterward. Late toxicities were defined as symptoms first occurring > 90 days after the completion of radiotherapy, or lasting > 90 days after the completion of radiation. Observed toxicities were graded and reported according to the toxicity criteria of the Radiation Therapy Oncology Group and the European Organization for Research and Treatment of Cancer [19]. The clinical conditions we followed included diarrhea, bowel movement, bleeding, obstruction, perforation fistula, and so on.

## Results

Based on the NIRS MKM percentage volume constraints, the converted LEM doses by our RBE-conversion model [12] (versus these converted by the CNAO’s study) were D20% ≤ 43.14 Gy (RBE) versus 42.90 Gy (RBE), D10% ≤ 58.48 Gy (RBE) versus 57.70 Gy (RBE), D5% ≤ 68.68 Gy(RBE) versus 68.20 Gy(RBE), and D0% ≤ 73.09 Gy(RBE) versus 72.00 Gy(RBE). The converted results were similar to those reported by the CNAO’s study.

Table 2 shows the MKM rectum constraints of percentage volume for 16-fraction CIRT and the converted LEM constraints for 16-, 12-, 8-, and 4-fraction CIRT, using both strategies. CNAO also proposed absolute volume constraints for clinics. Table 3 shows how the CNAO constraints for 16-fraction CIRT were converted to constraints for 12-, 8-, and 4-fraction CIRT using our two strategies.

**Table 2:** The MKM rectum constraints for 16-fraction CIRT and the converted LEM constraints for 16-, 12-, 8-, and 4-fraction CIRT from two strategies

^d^D_MKM_16fx	^e^D_LEM_16fx	^a^D_LEM_ 12fx			^b^D_LEM_ 8fx			^c^D_LEM_ 4fx		
		^f^MKM LQ	^g^LEM LQ	^h^Diff	MKM LQ	LEM LQ	Diff	MKM LQ	LEM LQ	Diff
D20% ≤ 28.80	43.14	37.60	39.55	5.18%	30.40	34.60	13.82%	20.80	26.83	28.98%
D10% ≤ 46.40	58.48	49.74	53.08	6.72%	39.25	45.86	16.83%	25.66	34.96	36.24%
^i^D5% ≤ 56.00	65.11	55.27	58.91	6.58%	43.41	50.69	16.76%	28.33	38.42	35.59%
^j^D0% ≤ 60.80	68.33	58.01	61.73	6.04%	45.46	53.03	14.27%	29.64	40.10	26.08%

^a^The LEM rectum constraints for 12-fraction CIRT [Gy (RBE)]

^b^The LEM rectum constraints for 8-fraction CIRT [Gy (RBE)]

^c^The LEM rectum constraints for 4-fraction CIRT [Gy (RBE)]

^d^The MKM rectum constraints for 16-fraction CIRT [Gy (RBE)]

^e^The LEM rectum constraints for 16-fraction CIRT from our previous study [Gy (RBE)]

^f^The LEM rectum constraints converted from MKM LQ strategy

^g^The LEM rectum constraints converted from LEM LQ strategy

^h^Difference = (LEM LQ-MKM LQ)/MKM LQ*100%

^i^Based on the publication [17], NIRS used D5% ≤ 56.00 Gy (RBE) as their constraints

^j^Based on the publication [17], NIRS used D0% ≤ 60.80 Gy (RBE) as their constraints

**Table 3:** CNAO LEM rectum constraints for 12-, 8-, and 4-fraction CIRT converted based on two strategies

^d^D_LEM_16fx	^e^D_MKM_ 16fx	^a^D_LEM_ 12fx			^b^D_LEM_ 8fx			^c^D_LEM_ 4fx		
		^f^MKM LQ	^g^LEM LQ	^h^Diff	MKM LQ	LEM LQ	Diff	MKM LQ	LEM LQ	Diff
D_10cc_ ≤ 54.00	39.99	45.97	49.14	6.90%	36.53	42.59	16.58%	23.94	32.60	36.19%
D_5cc_ ≤ 61.00	49.84	51.70	55.30	6.96%	40.73	47.70	17.12%	26.62	36.28	36.30%
D_1cc_ ≤ 66.00	57.31	55.97	59.69	6.65%	43.97	51.33	16.75%	28.67	38.88	35.59%

^a^The LEM rectum constraints for 12-fraction CIRT [Gy (RBE)]

^b^The LEM rectum constraints for 8-fraction CIRT [Gy (RBE)]

^c^The LEM rectum constraints for 4-fraction CIRT [Gy (RBE)]

^d^The CNAO rectum constraints for 16-fraction CIRT of absolute volumes [Gy (RBE)]

^e^The MKM constraints which were backward converted from CNAO rectum constraints based on our RBE-conversion model [Gy (RBE)]

^f^The LEM rectum constraints converted from CNAO constraints by MKM LQ strategy

^g^The LEM rectum constraints converted from LEM LQ strategy

^h^Difference = (LEM LQ-MKM LQ)/MKM LQ*100%

Table 4 lists the DVH parameters for the 38 patients receiving 16-fraction CIRT. Eight out of 38 patients slightly exceeded the D20% constraints, and four out of eight patients slightly exceeded the D10% constraints (see the dark gray in Table 4). The DVH parameters for two patients who received 12-fraction CIRT were within the LEM constraints for the 12-fraction CIRT using the MKM LQ strategy.

**Table 4:** DVH parameters from 40 patients versus the LEM constraints

Constraints (16fx)	^a^LEM constraints	^b^DVH summary	
		^c^Median	^d^Maximum
Our previous study	D20% ≤ 43.14	37.91	^e^49.96
	D10% ≤ 58.48	53.53	^f^62.12
	D5% ≤ 65.11	59.96	65.18
	D0% ≤ 68.33	62.91	66.61
CNAO’s study	D_10cc_ ≤ 54.00	37.58	53.52
	D_5cc_ ≤ 61.00	53.43	60.30
	D_1cc_ ≤ 66.00	62.54	65.86
Constraints (12fx)	^g^LEM Constraints	Patient 1	Patient 2
Constraints from MKM LQ	D20% ≤ 37.60	24.27	30.53
	D10% ≤ 49.74	38.65	46.75
	D5% ≤ 55.27	46.45	52.86
	D0% ≤ 58.01	54.11	55.37
	D10cc ≤ 45.97	29.89	27.10
	D5cc ≤ 51.70	42.17	44.55
	D1cc ≤ 55.97	53.20	54.80

^a^LEM rectum constraints for 16-fraction CIRT, the percentage volume constraints were from our previous study, the absolute volume constraints were from CNAO [Gy (RBE)]

^b^The value of D20%, D10%, D5%, D0%, D_10cc_, D_5cc_, and D_1cc_ parameters processed from each patient’ rectum DVH of 38 patients who received 16-fraction CIRT and 2 patients who received 12-fraction CIRT [Gy (RBE)]

^c^The median value of DVH parameters among the 16-fraciton group and 12-fraction group [Gy (RBE)]

^d^The maximum value of DVH parameters among the 16-fraciton group and 12-fraction group [Gy (RBE)]

^e^Eight patients were over the D20% constraints for 16-fraction CIRT

^f^Four out of 8 patients were over the D10% constraints for 16-fraction CIRT

^g^The LEM rectum constraints converted from MKM LQ strategy for 12-fraction CIRT

**Fig. 2:**
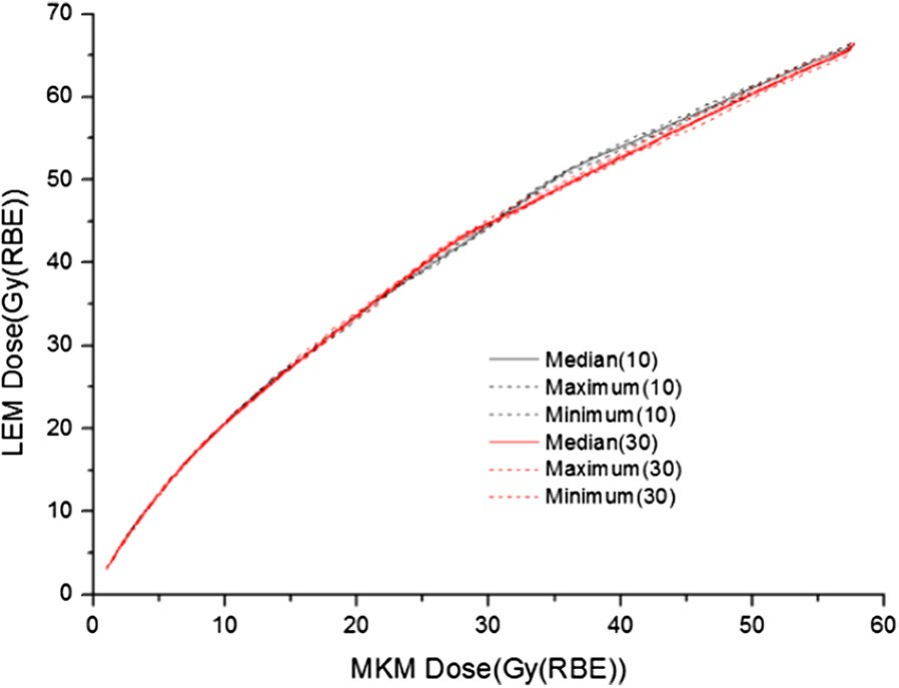
The 16-fraction conversion curves; the black curve is based on 10 patients and the red curve is based on 30 patients

Until Aug 2020, and after the median follow-up (MTF) of 10.8 months (7.1–20.8), none of the patients reported ≥ G1 late rectum complications. Meanwhile, no patients reported experiencing diarrhea, constipation, bleeding, perforation, stricture, or pain. For the 16-fraction CIRT, the differences between the RBE-conversion model based on 10 patients and the model based on 30 patients were − 0.62% (− 3.02% to 2.49%). Detailed information is presented in Fig. 2, Appendix.

## Discussion

Two strategies were used to convert the MKM rectum constraints for 16-fraction CIRT to the LEM rectum constraints for 12-, 8-, and 4-fraction CIRT. Our results indicated up to a 36.30% difference. The RBE-conversion model conversions based on 10 patients were similar to the conversions based on 30 patients. The follow-up data show that no patient reported ≥ G1 late rectum complications.

Former 20- to 12-fraction CIRT with MKM indicated that the shorter CIRT course could greatly reduce treatment costs while maintaining the same clinical outcomes [3–5, 20]. Therefore, for the benefit of patients seen at our center, we used LEM during a new clinical trial of 12-fraction CIRT for patients with PCA to escalate the prescription from 54.00 Gy (RBE) to 58.80 Gy (RBE). To prepare for this new study, we sought to derive the rectum constraints for this protocol. If our clinical trial eventually validates the feasibility of the converted rectum constraints, it may be possible to convert other OAR constraints using these strategies to achieve a shorter CIRT.

The 16-fraction NIRS-based LEM constraints from CNAO’s study and our converted results based on the RBE-conversion model were similar. Thus, we could perform our MKM LQ strategy on the CNAO constraints. The main difference between two strategies was the conversion factors (CFs) applied for RBE conversion. NIRS validated the transferability of the prescription from 20- to 12-fractions with a LQ model, but in MKM. To follow NIRS success, we should use a backward approach to convert CNAO constraints to MKM. However, since the endpoint of our study was to use their experience for our LEM-based CIRT, a subsequent re-conversion from MKM to LEM was necessary. Our previous study was based on only 10 patients. To investigate whether more patients could improve the accuracy of the RBE-conversion model, we tested our model using another 30 patients. However—since the 10- and 30-patient approaches produced similar results—we still used the RBE-conversion model based on 10 patients. The difference between the models using 10 or 30 patients revealed the uncertainty of our approach. Furthermore, we expect little difference for applying a 16-fraction RBE-conversion model to 12-, or even 8-fraction CIRT. However, we may need to re-generate the RBE-conversion model for 4-fraction CIRT. In our clinical practice, we are using the LEM rectum constraint DVH from our previous study (e.g., D3cc ≤ 60 Gy (RBE)) for 16-fraction CIRT. Meanwhile, we continue to explore 12-fraction constraints, but using percentage volume constraints from the MKM LQ strategy as the starting constraints.

Takakusagi et al. [20] published the preliminary results of CIRT using spot scanning for treating PCAs using a 12-fraction protocol. This fractionation was similar to the NIRS apparatus [3] and our new clinical trial. The spot-scanning delivery was the same as ours. The five-year results of the Takakusagi’s study independently verified the NIRS results, demonstrating that the 12-fraction protocol had similar clinical outcomes and OAR complications as the 16-fraction CIRT. Besides, the prescription and rectum constraints in their 12-fraction CIRT study supported the feasibility of our approach. In their study, the rectum constraint was given as V80% < 10 cc and the prescription was 51.6 Gy (RBE)/12fx. Based on our RBE-conversion model for 12-fraction CIRT, the corresponding LEM constraint and prescription were 49.00 Gy (RBE) < 10 cc and 55.71 Gy (RBE)/12fx (4.64 Gy (RBE)/fx). These parameters were similar to the D10cc < 49.14 Gy (RBE) converted from CNAO LEM constraints using LEM LQ strategy (see Table 3) and our clinical prescription for 12-fraction CIRT. These results gave us confidence in the future of this method.

Based on the LEM rectum constraints for 12-fraction CIRT and the Japanese experience with 12-fraction CIRT, a proposed clinical procedure for performing the 12-fraction CIRT is that: use the LEM constraints from MKM LQ strategy as the starting constraints; as the prescription is escalated—and if a patient’s rectum dose was more than the LEM constraints from the MKM LQ strategy but lower than the LEM LQ strategy—this patient could still receive CIRT; however, during treatment and post-treatment follow-up, physicians may closely monitor rectum complications. Importantly, complications are crucial for adapting the constraints; for a patient whose rectum doses exceed the constraints from the LEM LQ strategy, the physician may ask the patient to receive another treatment first (e.g., anti-hormonal therapy until the prostate is small enough so the rectum dose is lower than the LEM constraints from the LEM LQ strategy).

Our 16- and 12-fraction LEM rectum constraints require further validation based on a longer follow-up period. NIRS experience has shown that 81% of the late rectum toxicities occurred within 24 months after CIRT. While our current MTF was only 10 months. The LEM rectum constraints in this study for CIRT with < 16-fraction protocols are based on clinical experience with 16-fraction CIRT. Our limited follow-ups showed more-frequent complications related to urethral overdoses from the 12-, compared to the 16-fraction, protocol. This suggested that the 16- and 12-fraction CIRT may not be equivalent. The translation from 16-fraction CIRT to smaller fractionation CIRT is based on a LQ model and an RBE-conversion model. NIRS only validated the MKM prescription transferability from the 20- to the 12-fraction protocol based on a LQ model, not including the rectum constraints. The LEM experience with the skull-based chordomas suggested the constraint conversions based on a LQ model could be used but with caution [21]. And the strategy (i.e., MKM LQ or LEM LQ) effectiveness still remains an open question. In addition, NIRS applied a one-beam-per-day, four-fraction-treatment-per-week protocol. Our center applies a two-beam-per-day, five-fraction-treatment-per-week protocol. More beams per week may lead to more-frequent complications. To address these questions, a study based on longer follow-up time and more patients is needed in the future.

## Conclusions

Two strategies were established to convert NIRS and CNAO rectum constraints for 16-fraction CIRT to the LEM constraints for 12-, 8-, and 4-fraction CIRT in patients with PCA. Significant differences were found in the converted constraints. We believe these differences were due to the CFs applied for RBE conversion. Our limited clinical follow-up from the patients who received 16- and 12-fraction CIRT showed that—for the sake of safety—the LEM rectum constraints from the MKM LQ strategy for PCAs could be used as starting constraint reference for the respective hyperfractionated CIRT. However, these results are preliminary. Due to the relatively small patient cohort and limited follow-up duration, additional studies are indicated.

## Data Availability

The datasets used and analyzed during the current study are available from the corresponding author on reasonable request.

## Appendix

Figure 2 shows the conversion curves of the RBE-conversion model for 16-fraction CIRT, the black curve is based on 10 patients, and the red curve is based on 30 patients. Two conversion curves are matched with each other. Based on the median value, the difference between these from 10 patients and these from 30 patients was − 0.62% (− 3.02% to 2.49%).

## Abbreviations

MKM: Microdosimetric kinetic model
CIRT: Carbon-ion Radiotherapy
LEM: Local effect model
PCA: Prostate carcinoma
LQ model: Linear-quadratic model
CNAO: Centro Nazionale di Adroterapia Oncologica (CNAO), Italy
NIRS: National Institute of Radiobiological Science, Japan
16fx: 16-Fraction CIRT
12fx: 12-Fraction CIRT
8fx: 8-Fraction CIRT
4fx: 4-Fraction CIRT
MKM dose: RBE-weighted doses with MKM
LEM dose: RBE-weighted doses with LEM
OAR: Organ at risk
